# Does Sports Industry Matter in Human Wellbeing: Evidence From China?

**DOI:** 10.3389/fpubh.2022.872506

**Published:** 2022-03-24

**Authors:** De Ping Wang, Juan Lin

**Affiliations:** Wuchang University of Technology, Wuhan, China

**Keywords:** sports, tourism, health, ARDL, China

## Abstract

It is widely considered that sport and physical activities contribute to the development of human wellbeing. It is a fact that sport brings positive energy, discipline, and human wellbeing. Sports have an enormous effect on human health. Therefore, we assess the effects of the sports industry on the human health of China by using the autoregressive distributed lag (ARDL) approach from 1998 to 2020. Findings show that sports activities significantly improve human health and wellbeing. Tourism has found a positive influence on health and helped to contribute to human wellbeing. Empirical results prove that health expenditure and financial development significantly increase the population health in China. China's government should focus on the sports and tourism industry to play an important role in human health and wellbeing.

## Introduction

Health is not just the absence of disease but indicates people's physical, social, and mental wellbeing. The demand for good health facilities also rises due to increasing people's living standards. Public health has become the most important policy concern for policymakers and governments all around the globe. Policymaking in the domain of public health includes various methods that require extensive research and resources, which are necessary to protect society from diseases and improve the life expectancy of the people (1). Indeed, policies in the field of the health sector are not easy to comprehend and implement. Douglas et al. (2) also highlighted the complication in implementing health policies due to a wide variety of scrutinizing and evaluation approaches, a series of interventions, and different types of health professionals performing their duties in a diverse environment (3).

In recent times, physical activity has become a determinant of health quality. Various researchers have highlighted many health-related threats attached to a lack of physical activity. Furthermore, they have also pointed out many health benefits that can be attained through continuous and regular involvement in physical activities and sports. Most of these works have been performed in the field of medical and behavioral sciences. Nevertheless, some recent works have identified that the relationship between sport, physical activities, and health outcomes is quite complex in nature (4). Few other empirics have pointed out the policy dimensions and commercial activities related to sport and its marketing dimensions (5). Some researchers have discovered a relationship between sports medicine and health and explored that this domain of sports medicine is quite complicated (6). In contrast, few others have targeted the mechanism involved in comprehending the complex processes, practices, and health concerns related to sport and physical activities (6, 7).

The association between sport, physical activity, and health status is not easy to understand but represents complicated, complex sequential and spatial efforts regarding political strategies, social ideologies, policy designs, and policy implementations. In the light of the above discussions, a few other issues, such as aging, infirmity, socio-economic position, culture, and gender, in the context of sports-health nexus has come to the fore [see, for example (8–12)]. To date, many studies are available in the disciplines of social gerontology, sport sociology, policy science and studies, gender studies, and international studies that have tried to explore the link between physical activity and health status, but still, there is much room available in this field to have an explicit discussion which can help us to understand the complex nature of politics and policy measures involved in sport, physical activity, and public health (13). Maugeri et al. (14) noted that Physical activity has a positive impact on psychological health and human wellbeing. A similar finding is also reported by Kekäläinen et al. (15), who found that physical activity improves subjective health and mental wellbeing.

In recent years, physical activities, including sports, have increased sharply (16). But Brownson et al. (17) added that these activities include a very little portion of daily basis physical activities. The decline in physical activities occurs due to technological, environmental, social, and economic changes. The decline in physical activities results in an upsurge of other activities such as video gaming, television, computers, hand-held consoles, and tablets (18). Other activities of daily routine such as occupational, transport, domestic, and leisure time have undertaken physical activities (19). Moreover, physical activities at the domestic level are also declining due to the increased role of technology in household chores. The reduction in physical activities and sports results in declining public health (20). Thus, there is a need to understand the determinants that can engage people in sports and physical activities. This initiative will promote a sports culture that would lead to better approaches for attaining public health and physical activity recommendations.

From a policy perspective, it is fundamental that sports programs ensure the benefits of physical welfare and deliberately come up with desired outcomes (21, 22). Sports are conserved as a supplement for the goodness of health and wellbeing (23). The literature argues that sports act as a dominant social institution (24). It is suggested that the sports industry is required for personal competence, skills development, enjoyment, and diversification of activities (25, 26). Hence, the imperative role of sports cannot be denied in the healthy development of any society. Sports contribution is highly regarded in the field of human health and wellbeing. In this perspective, our study is a pioneer in determining the crucial role of the sports industry on human health and wellbeing. China is selected based on data availability. China's sports industry continues to grow. The China sports industry market size was estimated to touch 3 trillion yuan. This study aims to explore the impact of the sports industry on human health and wellbeing in the case of China over the period 1998–2020. The study employed Autoregressive Distributed Lag (ARDL) approach for empirical tasks. No previous study has examined the impact of the sports industry on human wellbeing in the short-run and long-run in the context of China. The study is a move toward starting a new debate regarding the sports industry on human wellbeing in the field of health economies. Previous research work ignored the transmission channels among sports industry on human wellbeing, while our study provides effects and transmission channels in empirical analysis. The findings of the study will support policymakers in designing such policies that engage individuals in sports and physical activities that ultimately would be beneficial in improving their physical fitness.

## Methodology And Data

Sports have an enormous effect on an individual's daily life and human health. Physical activities like sports increase heart function, control blood sugar, reduce the risks of diabetes, and lower stress and tension levels. Several other macroeconomic variables, such as tourism, health expenditure, the internet, and financial development, are assumed to affect human wellbeing. From the literature and more precisely, the model by Bloodworth et al. (27) and Mansfield and Piggin (5) is adopted to explain the nexus between the sports industry and human health. The basic model can be represented as:


(1) $$\begin{array}{l} \text{HD}{\text{I}}_{\text{ t}}=\;\delta_{0}+\;\delta_{1}\text{Sport}{\text{s}}_{\text{t}}+\delta_{2}\text{Touris}{\text{m}}_{\text{t}}+\delta_{3}\text{H}{\text{E}}_{\text{t}}+\delta_{4}\text{Interne}{\text{t}}_{\text{t}}\\ +\;\delta_{5}\text{F}{\text{D}}_{\text{t}}+\varepsilon_{\text{t}} \end{array}$$

Where HDI is the human development index; Sports the sports industry; Tourism is tourism industry; HE is the health expenditure; the Internet is internet users; FD is financial development, ε_*t*_ the white noise error term, and δ_0_ the constant term. Human wellbeing is affected by the right-hand side variables in similar ways. From the standard literature, our estimates of δ_1_, δ_2_, δ_3_, δ_4_, *and δ*_5_ could be positive increases in physical activities, health expenditure, information, and financial resources, increasing human wellbeing. Estimation of Equation (1) yields only long-run estimates. Thus, an error-correction model needs to be specified using the ARDL model to incorporate the short-run effect. The ARDL econometric approach was formerly announced by Pesaran and Shin (28) and later augmented by Pesaran et al. (29). The error-correction model can be written as:


(2) $$\begin{array}{l} \Delta {\text{HDI}}_{\text{t}}=\delta_{0}+\sum_{\text{k=1}}^{\text{n}}\eta_{\text{1k}}\Delta {\text{HDI}}_{\text{t}-\text{k}}+\sum_{\text{k=0}}^{\text{n}}\eta_{\text{2k}}\Delta {\text{Sports}}_{\text{t}-\text{k}}\\ \;+\sum_{\text{k=1}}^{\text{n}}\eta_{\text{3k}}\Delta {\text{Tourism}}_{\text{t}-\text{k}}+\sum_{{}_{\text{k=0}}}^{\text{n}}\eta_{\text{4k}}\Delta {\text{HE}}_{\text{t}-\text{k}}\\ \;+\sum_{\text{k=1}}^{\text{n}}\eta_{\text{5k}}\Delta {\text{Internet}}_{\text{t}-\text{k}}+\sum_{{}_{\text{k=0}}}^{\text{n}}\eta_{\text{6k}}\Delta {\text{FD}}_{\text{t}-\text{k}}\\ \;+\delta_{1}{\text{HDI}}_{\text{t}-\text{1}}+\delta_{2}{\text{Sports}}_{\text{t}-\text{1}}+\delta_{3}{\text{Tourism}}_{\text{t}-\text{1}}\\ \;+\delta_{4}{\text{HE}}_{\text{t}-\text{1}}+\delta_{5}{\text{Internet}}_{\text{t}-\text{1}}+\delta_{6}{\text{FD}}_{\text{t}-\text{1}}\\ \;+\delta .{\text{ECM}}_{\text{t}-\text{1}}+\varepsilon_{\text{t}} \end{array}$$

The error-correction Equation (2) is due to Pesaran et al. (29), where the short-run effects reflected by the η_1k_, η_2*k*_, η_3*k*_, η_4*k*_, η_5*k*_, and η_6k_. The long-run effects of variables are reflected in the estimates of δ_2_, δ_3_, δ_4_, δ_5_, *and δ*_6_ normalized on δ_1_. The ARDL approach is based on the joint F-statistics for cointegration analysis that uses new critical values. The null hypothesis of cointegration among the variables is (Ho: δ_1_ = δ_2_ = δ_3_ = δ_4_ = δ_5_ = δ_6_= 0) against the alternative hypothesis (H1: δ_1_ ≠ δ_2_ ≠ δ_3_ ≠ δ_4_ ≠ δ_5_ ≠ δ_6_= 0). Unlike other time series econometric techniques, the ARDL does not limit the model to employ the same order of integration. This means that we can apply ARDL at mixed order of integration. Secondly, other time series cointegration approaches are more sensitive to the sample size, while the ARDL is more suitable in the case of small sample size (30, 31). Lastly, we follow the Hendry (32) general-to-specific econometric approach to select a parsimonious model as possible and permits the diagnostic tests: (i) Lagrange Multiplier (LM) test for serial correlation; (ii) Breusch–Pagan test for heteroscedasticity; (iii) Ramsey RESET test for model misspecification; and (iv) stability tests of CUSUM and CUSUM of squares.

The study intends to explore the effect of the sports industry on human health and wellbeing in the case of China. The study adopted time-series data set for the period 1998 to 2020 for empirical investigation. The details regarding symbols of variables and definitions are given in Table 1. Life expectancy determines health outcomes, while the human development index determines human wellbeing. A similar approach is also adopted by Majeed (33). Sports industry role is measured by using two proxies, namely sports & entertainment and the sports industry market size. The study has used some control variables in the analysis as well. These are international tourism, current health expenditures as a percent of GDP, individuals using the internet as a percent of the population, and financial development index. The required data for this study has been scrutinized by the World Bank, UNDP, National Bureau of Statistics of China, and IMF. The analysis of descriptive statistics is provided in Table 1.

**Table 1:** Variables definition and data description.

**Variables**	**Definitions**	**Mean**	**Median**	**Maximum**	**Minimum**	**Std. Dev**.	**Skewness**	**Kurtosis**
LE	Life expectancy at birth, total (years)	74.1	74.11	77.36	70.73	2.008	−0.057	1.821
HDI	Human development index	0.687	0.694	0.796	0.576	0.068	−0.122	1.804
SE	Sport and entertainment (RMB bn)	4.875	3	6.752	2.154	1.458	0.657	2.143
SPORTS	Size of the sports industry market (trillor yuan)	1.098	0.753	2.674	0.36	0.763	1.03	2.599
TOURISM	International tourism, number of arrivals	18.59	18.67	18.92	17.96	0.255	−0.946	3.088
HE	Current health expenditure (% of GDP)	4.517	4.473	5.351	3.659	0.433	0.017	2.341
INTERNET	Individuals using the Internet (% of population)	29.16	28.9	70.64	0.169	23.49	0.196	1.581
FD	Financial development index	0.507	0.521	0.668	0.344	0.102	−0.027	1.674

## Empirical Results And Discussion

Empirical findings of regression models and preliminary tests are given in this section. Table 2 provides empirical outcomes of unit root tests. The findings of the DF-GLS test report that sport & and entertainment and sports are level stationary variables. At the same time, life expectancy, human development index, tourism, health expenditure, use of the internet, and financial development are the first difference stationary variables. The findings of the PP test display that tourism is a level stationary variable while all other variables are first difference stationary. Based on the GF-GLS test and PP test findings, the study employed the ARDL estimation technique for regression analysis. Table 3 reports the long-run and short-run results of health and human being models.

**Table 2:** DF-GLS and PP tests.

	**DF-GLS**		**PP**		**Decision**	
	**I(0)**	**I(1)**	**I(0)**	**I(1)**	**DF-GLS**	**PP**
LE	1.254	−2.615^**^	−1.235	−2.857^*^	I(1)	I(1)
HDI	−0.123	−4.235^***^	−0.254	−4.587^***^	I(1)	I(1)
SE	−1.859^*^		0.231	−2.687^*^	I(0)	I(1)
Sports	−2.355^**^		2.212	−2.802^*^	I(0)	I(1)
Tourism	−0.133	−3.566^***^	−3.256^**^		I(1)	I(0)
HE	−0.785	−3.754^***^	−0.755	−3.687^***^	I(1)	I(1)
Internet	0.123	−1.654^*^	0.125	−2.654^*^	I(1)	I(1)
FD	0.258	−4.665^***^	0.285	−4.687^***^	I(1)	I(1)

*** *p < 0.01*;

** *p < 0.05*;

* *p < 0.1*.

**Table 3:** Short and long-run estimates of health and human wellbeing.

**Variable**	**Health**		**HDI**	
	**Coefficient**	***t*-Stat**	**Coefficient**	***t*-Stat**
**Short-run**				
SPORTS	0.578^***^	3.497	0.020^**^	2.798
SPORTS(-1)	0.140	0.549		
SPORTS(-2)	0.615	1.640		
TOURISM	0.636^**^	2.343	0.011	0.610
TOURISM(-1)	0.838^**^	2.136	0.046^**^	2.574
TOURISM(-2)	0.601	1.498	0.035	1.571
HE	0.216^**^	2.218	0.016^**^	2.117
HE(-1)	0.224^***^	3.553	0.017^***^	3.249
HE(-2)	0.123	1.022	0.016^**^	2.231
INTERNET	0.001	0.145	0.002^***^	3.537
INTERNET(-1)	0.016	0.763	0.003^***^	2.637
INTERNET(-2)	0.020	1.348		
FD	0.279	0.482	0.067^*^	1.724
FD(-1)	1.135^**^	1.969	0.044	0.942
**Long-run**				
SPORTS	0.495^*^	1.871	0.018^*^	1.917
TOURISM	5.785^***^	3.954	0.161^**^	2.194
HE	1.570^*^	1.723	0.054	1.077
INTERNET	0.006	0.269	0.000	0.155
FD	3.942^**^	2.346	0.187^**^	1.983
C	3.253	1.382	2.630	1.685
**Diagnostics**				
F-test	7.895^***^		7.012^***^	
ECM(-1)^*^	−0.359^***^	9.620	−0.411^***^	8.593
LM	1.254		1.452	
BP	1.654		0.125	
RESET	0.524		0.745	
CUSUM	S		S	
CUSUM-sq	S		S	

*** *p < 0.01*;

** *p < 0.05*;

* *p < 0.1*.

In Table 3, long-run findings show that the impact of sports is significant and positive on health and human wellbeing, implying that the sports industry contributes significantly to improving human health and wellbeing. It is found that a 1 percent upsurge in the sports industry improves human health by 0.495 percent and increases human wellbeing by 0.018 percent in the long-run. This finding is consistent with Mansfield and Piggin (5), who noted that sport has an essential role play in health and wellbeing. The finding is also supported by Bloodworth et al. (27), Cairney et al. (34), and Malm et al. (35). They infer that the sport and entertainments industry has numerous direct and indirect health gains through its favorable impact on heart disease, hypertension, blood sugar, risks of diabetes, and healthy muscles. This finding is also supported by Snedden et al. (36), who noted that sport and a higher level of physical activity are linked with more positive human health. The finding is also backed by Pascoe et al. (37), who noted that increased physical activity increases human health. This means that sports and physical activity improve a healthy lifestyle by reducing harmful activities of human health. Sport improves diet, discourages alcohol and drugs, helps reduce violence, and promotes social networking, thus increasing human health. World Health Organization (2003) noted that sport is the most cost-effective and sustainable way to reduce health problems. Sport promotes psychological wellbeing by reducing stress and increasing happiness.

Tourism reports a significant and positive influence on human health and wellbeing. It shows that a 1 percent escalation in tourism activities tends to improve health by 5.785% and increases human wellbeing by 0.161% in the long-run. Health expenditure and health outcomes are significantly and positively associated in the long-run, but no significant association is found between human wellbeing and health expenditures. The findings show that 1 percent intensification of health expenditures results in improving health outcomes by 1.570% in the long-run. No significant association is found between internet use, health outcomes, and human wellbeing in the long-run. In contrast, financial development reports a significant and positive impact on health outcomes and human wellbeing, revealing that access to finance improves households' health and living standards in the long-run. The coefficient estimates display that a 1 percent upsurge in financial development increases health quality by 3.942% and human wellbeing by 0.187% in the long-run.

The findings report that sports are significantly and positively associated with human health and wellbeing in the short-run. Tourism reports a significant and positive impact on human health and a statistically insignificant impact on human wellbeing in the short-run. However, health expenditure is positively linked with human health and human wellbeing in the short-run. Internet and financial development both variables report significant and increasing impacts on human wellbeing but statistically insignificant impact on human health in the short-run.

The study has confirmed the robustness of empirical findings through a variable-based method. The study has used sports and entertainment as a focused variable for robustness testing. The findings of robust models are given in Table 4. The coefficient estimates of robust models display those sports and entertainment are positively associated with health and human development, revealing that sports and entertainment improve health quality and human wellbeing in the long and short-run. It shows that a 1% escalation in sports and entertainment increases health quality by 0.015% and human wellbeing by 0.007% in the long-run. In the short-run, 1% escalation in sports and entertainment reports a 0.011% rise in health quality and a 0.009% rise in human wellbeing. Some important diagnostic tests have been performed to confirm the validity of regression results. The coefficient estimates of these tests are given in the lower panels of both tables. The F-stat and ECM test findings confirm the existence of long-run cointegration among variables. The coefficient estimates of the LM test and BP test confirm that no autocorrelation and heteroskedasticity are reported in all models. All models are correctly specified, as shown by the findings of the Ramsay RESET test. Stability conditions hold in all models as demonstrated by the CUSUM and CUSUM-sq tests findings.

**Table 4:** Short and long-run estimates of health and human wellbeing (Robustness).

**Variable**	**Health**		**HDI**	
	**Coefficient**	***t*-Stat**	**Coefficient**	***t*-Stat**
**Short-run**				
SE	0.011^***^	3.357	0.009^***^	2.773
SE(-1)	0.007^**^	2.481	0.004	1.276
SE(-2)	0.001	1.033	0.001	1.194
TOURISM	0.112	0.800	0.017	0.898
TOURISM(-1)			0.085^***^	3.438
TOURISM(-2)			0.076^**^	2.584
HE	0.011	0.264	0.022^**^	2.373
HE(-1)	0.155^***^	3.837	0.028^***^	3.952
HE(-2)			0.032^***^	2.706
INTERNET	0.016^***^	3.214	0.004^***^	3.116
INTERNET(-1)	0.022^***^	3.529	0.004^***^	2.778
FD	0.757^***^	2.638	0.071^*^	1.702
FD(-1)	0.101	0.350	0.135^**^	2.051
FD(-2)	0.571^*^	1.916	0.081^*^	1.718
**Long-run**				
SE	0.015^***^	2.906	0.007^**^	2.228
TOURISM	0.840	0.956	0.239^***^	3.472
HE	1.080^**^	2.063	0.110^**^	2.214
INTERNET	0.043^***^	3.746	0.002	0.834
FD	2.153	0.747	0.386^***^	2.940
C	6.700^***^	3.391	4.383^***^	2.910
**Diagnostics**				
F-test	6.654^***^		7.218^***^	
ECM(-1)^*^	−0.433^***^	9.044	−0.543^***^	8.059
LM	1.325		0.356	
BP	1.255		1.25	
RESET	1.258		1.023	
CUSUM	S		S	
CUSUM-sq	S		S	

*** *p < 0.01*;

** *p < 0.05*;

* *p < 0.1*.

## Conclusion And Implications

Sports tend to have positive and negative impacts on health. Positive benefits of health are obtained through physical activity. Sports bring health benefits through personal development, psychological development, and reduction in alcohol consumption. Knowledge about health, exercise, and nutrition can be obtained through sports activities. However, sports bring negative impacts on health in the form of risk that leads to poor mental condition, eating disorders, discomfort related to the gastrointestinal issue, burnout, and the risk of injury. Sports are more likely attached to psychological and physical abuse that negatively influences human health. The role of sports in every society has increased significantly; thus, the impact of sports on health outcomes and human development cannot be ignored. In this perspective, our study makes an effort to explore the impact of sports on human health and human wellbeing in the case of China over the period 1998–2020. The study employed the ARDL approach for deducing empirical estimates. The study also tested the robustness of findings via the variable-based robustness method. The key findings of the study are described as follows. Sports are found to positively impact health and human wellbeing both in the long-run and short-run. Tourism reports a significant and positive effect on health and human wellbeing in the long-run and on health in the short-run. A significant and positive association is found between health quality and health expenditures in the long-run and short-run and between health expenditures and human wellbeing in the short-run only. Financial inclusion reports a positive influence on human health and wellbeing in the long-run and human wellbeing only in the short-run. The use of the internet influences human wellbeing only in the short-run. In robust models, sports and entertainment report significant and positive influences on human health and wellbeing in the long-run and short-run.

Our findings recommend healthy sports programs at the grassroots level in China. China's governments should increase physical activity in the context of human health. Authorities raise awareness in society about the multiple benefits of physical activity. National and provincial physical activity programs and initiatives should be effectively planned and coordinated with realistic and perfect objectives. Such planning will be arranged within a unified approach to sustainable socio-economic development and health promotion. Government should also increase sports ad health expenditure in the economy to achieve the health objectives. Educational institutions and media enhance awareness of physical activity and help promote human health. Human health policies, and more specifically physical activity policies, should underscore the role of sport in improving health and wellbeing.

This study undergoes several limitations. Life expectancy is used to measure human health while infant mortality, mental health, physical health, chronic diseases are ignored. The data unavailability restricted our empirical analysis for a short period. Further empirical research can be conducted using a large and up-to-date study dataset. Authors should also extend the analysis for developing economies by employing other indicators of human health. Future studies focus on other measures of physical activity and their effects on happiness, health, and human wellbeing.

## Data Availability Statement

Publicly available datasets were analyzed in this study. This data can be found at: https://data.worldbank.org.

## Author Contributions

DW: conceptualization, software, data curation, and writing-original draft preparation. JL: methodology, writing-reviewing, editing, visualization, and investigation. All authors contributed to the article and approved the submitted version.

## Conflict of Interest

The authors declare that the research was conducted in the absence of any commercial or financial relationships that could be construed as a potential conflict of interest.

## Publisher's Note

All claims expressed in this article are solely those of the authors and do not necessarily represent those of their affiliated organizations, or those of the publisher, the editors and the reviewers. Any product that may be evaluated in this article, or claim that may be made by its manufacturer, is not guaranteed or endorsed by the publisher.
